# Is the Prospective Link between Parental Stress and Adolescent Snack Intake or Weight Outcome Mediated by Food Parenting Practices?

**DOI:** 10.3390/nu13082485

**Published:** 2021-07-21

**Authors:** Maaike Koning, Jacqueline M. Vink, Carry Renders, Natascha Notten, Rob Eisinga, Junilla K. Larsen

**Affiliations:** 1 Knowledge Centre for Health and Social Work, Department of Healthy Society, Windesheim University of Applied Sciences, P.O. Box 10090, 8000 GB Zwolle, The Netherlands; n.notten@windesheim.nl; 2 Behavioural Science Institute, Radboud University, P.O. BOX 9104, 6500 HE Nijmegen, The Netherlands; jacqueline.vink@ru.nl (J.M.V.); rob.eisinga@ru.nl (R.E.); junilla.larsen@ru.nl (J.K.L.); 3 Department of Health Sciences, Vrije Universiteit, De Boelelaan 1105, 1081 HV Amsterdam, The Netherlands; carry.renders@vu.nl

**Keywords:** parental stress, food parenting, mediation analysis, adolescence, snacking

## Abstract

Parental stress may influence adolescents’ food intake and weight development over time, however, it is largely unknown why this is the case. This study examines whether the link between parental stress and adolescents’ snack intake and weight outcome is mediated by food parenting practices (FPPs). Participants included 400 parents and their adolescent children (aged 12–16) who completed questionnaires. The Perceived Stress Scale (PSS) was used to assess parental general stress levels and the Adolescent Food Parenting Questionnaire (AFPQ) to assess FPPs. Multiple mediation analyses with parallel mediators were performed, with parental general stress as an independent variable and adolescent snack intake and zBMI as dependent variables. FPPs (autonomy support, coercive control, modeling, healthy structure, snack structure) were entered as mediators in the model, adjusted for covariates. Autonomy support mediated the link between parental general stress and adolescent savory snack and sweet snack intake at follow-up. Parents who reported higher stress levels provided less autonomy support, which resulted in more adolescent snacking. None of the other FPPs mediated any link between parental stress and intake or weight outcome, and no significant indirect effects were observed with zBMI as an outcome variable. Further research should replicate this finding and may further examine underlying mechanisms.

## 1. Introduction

Adolescence is characterized as being one of the most complex transitions in the lifespan. During adolescence, physical, developmental, and social changes occur that can affect eating behaviors and nutritional health, making this a vulnerable period for overweight development, a critical public health issue, a critical public health issue [1,2,3,4,5,6,7,8]. In the transition from childhood to adolescence, dietary quality and physical activity generally decline and unhealthy dietary and sedentary habits are formed [1]. For example, fruit, vegetable, and milk consumption decreases [1], whereas the consumption of soft drinks and energy-dense snacks increases [1,2,3]. In the literature, snacks have been defined as foods eaten between meals and are typically identified as nutrient-poor and energy-dense (i.e., sweets, cookies, chips, sugar-sweetened beverages). In the last decades, adolescents’ intake of calories from energy-dense snacks has increased considerably [9], with snacking in-between meals currently amounting to a quarter of daily energy for youth in some European nations [10]. Adolescents who snack frequently more often have poorer quality diets and total energy intake, and also show other related health risks for excess weight gain [9,11,12].

Parents are an important influence on the development of children’s and adolescents’ snacking behavior [13,14], snack intake [15,16,17], and weight trajectories [18,19]. They are regulators, providers, and models of the home food environment [20], and may exert their influence through food parenting practices (FPPs). FPPs are defined as context-specific acts of parenting concerning food and eating to socialize children toward certain behavior [21,22], and are associated with child dietary intake, eating behaviors, and weight development [23]. A central framework to categorize diverse food parenting constructs distinguishes three higher-order dimensions in food parenting: structure, autonomy support, and coercive control [21,22,24]. Structured parenting practices are practices that may directly affect the child food environment and include setting boundaries around when, how much food, and what is available, providing rules and routines around eating and mealtimes, and restricting children’s exposure to unhealthy foods. Autonomy support involves positive and responsive child-centered practices such as teaching the adolescent child about nutrition or involvement of the adolescent child in the preparation, planning, or shopping of food. Both structured and autonomy supportive practices are generally linked to more positive intake and weight outcomes among children and adolescents. Coercive control is characterized by constraint and obtrusive parenting behaviors aimed at controlling adolescent children’s eating, emotion, or behavior, including food restriction, pressure, threats, and emotional and instrumental feeding, and is known to be associated with more unhealthy eating and higher adolescent BMI [25.

Recent growing evidence suggests that parental mental health issues (i.e., depressive symptoms and psychological stress) are positively associated with heightened risk of child and adolescent weight gain, obesity, and unhealthy weight-related behaviors, such as unhealthy eating, lack of physical activity, increased sedentary behavior, and unhealthy sleep patterns in children and adolescents [26,27,28,29,30,31,32,33,34,35,36]. Large-scale epidemiological studies have provided evidence that parental stress is linked to increases in children’s and adolescents’ Body Mass Index (BMI) [26,31,36]. Moreover, previous research has also shown that parental stress can impact parents’ FPPs [32,37,38,39,40,41,42,43]. Parents with higher general stress levels tend to be less responsive to children’s signs of hunger and satiation, and reported the use of more unhealthy coercive controlling food parenting strategies, particularly emotional and instrumental feeding [39,44,45,46,47]. Stressed parents were also more likely to manage children’s emotions or behavior by using food or snacks as coping strategies [48] and may also be more likely to snack more themselves [49] impacting children’s and adolescents’ snack intake through modeling and availability [49]. It is unknown whether parental stress is also associated with other FPPs. However, it is known that experienced stress may lead to short-term and easy solutions for acquiring and preparing food and meals [50,51]. As such, we suggest that parental stress may also influence other structured FPPs. Finally, parental stress may also negatively influence FPPs that support adolescent autonomy and encourage balance and variety around food, and provide nutritional education, due to time and cognitive restraints. In our obesogenic environment, parental stress may, thus, stimulate unhealthy food parenting, which, in turn, may impact adolescents’ snacking and weight development.

Although parental stress may impact diverse aspects of FPPs, certain FPPs may particularly influence adolescents’ development [52], given that adolescence is a time when youths develop the skills and characteristics that increase their autonomy. It is known that FPPs that support adolescent autonomy, manage variety and balance around food, and provide education about diet and nutrition [35]. Parenting that adjusts to the adolescent’s need for increased autonomy can promote the development of independence [53,54], and may encourage the adolescent’s development of healthful eating behaviors [55]. Structured practices may also be very important during adolescence. When parents provide a healthy food environment, set limits and rules, and establish healthy routines [20] that are associated with greater nutrition knowledge [56], unhealthy adolescent dietary intake may be prevented when adolescents are increasingly exposed to more diverse (unhealthier) environments [1,2,3]. We expect less strong links with coercive control as a mediator of the parental stress–FPPs link, as we observe reversed associations between adolescent weight outcomes and coercive practices, i.e., parents tend to adjust their controlling feeding practices in reaction to their child’s BMI rather than the opposite [57].

Moreover, evidence indicates that FPP use may depend on parental weight status or parent gender [58,59,60,61,62]. Notably, previous research has focused mainly on how mothers influence their children’s dietary habits, while less is known about the influence of fathers [58,63,64]. Moreover, fathers and mothers may respond to stress in different ways, which may influence the FPPs used. Children and adolescents with overweight or obese parents are more likely to become overweight themselves [65,66,67]. Moreover, parents with higher BMI themselves tend to use different (i.e., unhealthier) FPPs than parents with a healthy weight [68], and might particularly do so in response to stress [69].

### The Present Study

The present study examines an innovative model linking parental general stress to adolescent unhealthy eating behaviors (snacking) and zBMI between one and one and a half years later. Specifically, the study examines the mediating role of FPPs (i.e., autonomy support, coercive control, modeling, healthy structure, and snack structure) in this association by using one of the first food parenting measures for adolescents and their parents that has been developed (by the authors) and based on the content map of FPPs [22], the Adolescent Food Parenting Questionnaire (AFPQ). The AFPQ is relatively short, and can examine FPPs reported by parents as well as by adolescents [52]. We hypothesize that when parents experience a great deal of stress, they are less well capable of adopting healthy FPPs (because of time and cognitive restraints), which may be associated with unhealthier snacking behaviors and a higher zBMI in their adolescents. We expect that these mediating effects will particularly be found for those FPPs that are suggested to be important in adolescence, namely autonomy supportive FPPs and structured FPPs. In addition, we examine whether particular associations in the path model vary according to the parent’s gender and weight status, as the link between parental stress and FPPs may differ according to parents’ gender [70,71,72,73] and parental weight status [68,74].

## 2. Materials and Methods

### Participants and Procedure

The participants (i.e., both parents and their children) in the current study were part of the “G(F)OOD together” research project, a longitudinal study on adolescents’, and their parents’, health behavior in the Netherlands. Data for the first three waves were collected in fall 2017, spring 2018, and spring 2019, respectively. Adolescents and their parents were recruited through secondary schools. Dutch secondary schools are divided into three streams which represent different educational paths: one to prepare students for vocational training (‘VMBO’), a middle stream to prepare students to study at universities of applied sciences that focus on the practical application of arts and sciences (‘HAVO’), and another to prepare students for university (‘VWO’). Forty secondary schools in the south and the east of the Netherlands of different educational levels were approached randomly, to prevent selection bias, to participate in the cohort study. Six secondary schools agreed to participate in wave 1, and one additional school was added in wave 2. All adolescents attending the first and second grade and their parents were invited to participate in this study by means of an active parental consent procedure. Further details on the study design can be found elsewhere [75,76].

Parents provided written consent for themselves and their adolescents to participate in the study. Adolescents completed an online survey at school during one classroom hour (approximately 45 min), and height and weight were measured by trained research assistants. Parents completed an online questionnaire, which took approximately 20 min to complete. The questionnaires were administered through Qualtrics Survey Software (Qualtrics, Provo, UT, USA). Adolescents received a small gift after completing the questionnaire, and among participating parents several prizes were raffled. The Institutional Review Board of the Faculty of Social Sciences of the Radboud University, Nijmegen, The Netherlands approved the study protocol (reference number ECSW20170805-516) in 2017.

Parental consent was provided for 777 parents themselves and for 718 adolescents, of which 593 parents and 667 adolescents participated in the first wave. In total, 586 parents and 737 adolescents took part in wave 2, and 467 parents and 674 adolescents in wave 3. For the current study we used data from waves 1 through 3. We aimed to include data from both fathers and mothers. As not many fathers participated, we merged data from wave 1 and wave 2 as our baseline measure to enlarge the sample size and increase power (*n* = 675 parents). We were able to do so because both waves were collected within the same school year. Moreover, at wave 2 an extra school was recruited, for which wave 2 served as the baseline anyhow. Furthermore, we excluded non-biological parents for this study (*n* = 6), adolescents that were absent during the measurements (*n* = 33), adolescents of whom we had no matching parental data (*n* = 187) and, in cases where two caregivers participated (*n* = 49), we included the fathers to ensure the largest possible sample of fathers in the study sample, leaving a final sample of 277 biological mothers and 123 biological fathers (*n* = 400 parents). We used adolescent data from those adolescents of whom parents also had filled out questionnaires, to create parent–child dyads, resulting in a total of 400 parent–child dyads.

Most mothers (96.0%) and fathers (95.9%) were born in the Netherlands. Mean age of mothers was 44.6 years (SD_age_ = 4.2; age range = 29.8 to 57.3). Fathers’ mean age was 47.8 years (SD age = 4.1; age range = 37.8 to 61.4). Most parents had completed higher professional education (mothers: 44.0%; fathers: 41.5%) or secondary vocational education (mothers: 35.6%; fathers: 27.6%) and performed a paid job of less than 32 h per week (mothers: 63.8%; fathers: 21.5%) or 32 h per week or more (mothers: 21.5%; fathers: 74.4%).

Most adolescents were born in the Netherlands (97.5%), and boys (*n* = 197) and girls (*n* = 203) were approximately equally represented. All participants attended regular secondary education and were in their second or third year (M_age_ = 14.3 years; SD_age_ = 0.6; age range = 12.8 to 16.3) in wave 3. More than half of the participants (51.6%) were in pre-university education, 10.1% of the participants were in higher general secondary education, and 38.3% of the participants were in pre-vocational education.

## 3. Measures

### 3.1. General Perceived Stress

The 4-item Perceived Stress Scale (PSS) was used to assess parental general stress levels. The PSS is a self-report questionnaire, and is a global measure of stress that is easy to use. Many studies confirm its reliability and validity in a diversity of settings and in multiple languages [74,75,76,77,78,79]. The scale measures a person’s evaluation of stressful situations in the previous month of his or her life. The instrument consists of 4 statements that measure how uncontrollable and unpredictable respondents feel their lives are, for example: In the last month, how often have you felt confident about your ability to handle your personal problems? Respondents rate how often they experience stressful situations on a 5-point Likert scale ranging from ‘never’ to ‘very often’. Answers to the 4 items were summed into a total PSS score. The higher the score on the PSS, the more the respondent perceives that demands exceed the ability to cope. Cronbach’s alpha of the Perceived Stress Scale was 0.68 at baseline and 0.68 at follow-up (1 or 1.5 years later).

### 3.2. Food Parenting Practices

To assess FPPs we used the parental version of the Adolescent Food Parenting Questionnaire (AFPQ). This is a self-report questionnaire for parents of adolescents specifically. It consists of 16 questions concerning food parenting practices, for example: I have rules about when my child is allowed to eat snacks and how much (structure snacking); I sometimes give my child a small snack as comfort (coercive control); I discuss why it is important to eat fruit and vegetables with my child (autonomy support); There are always fruit and vegetables at home for my children to eat (healthy structure); I consciously eat vegetables or fruit when my child is around (modeling). For each item, answers could be given on a 5-point Likert scale ranging from “strongly disagree” (score 1) to “strongly agree” (score 5). Answers of the separate constructs (autonomy support, healthy structure, coercive control, snack structure, and modeling) were summed into a score per FPPs. The higher the score on the FPPs, the more it was put into practice.

### 3.3. Adolescents’ Snack Intake

To assess adolescents’ intake of sugar-sweetened beverages (SSBs), sweet snacks, and savory snacks, adolescents were asked to complete a food frequency questionnaire (FFQ). Specifically, the FFQ assessed participants’ intake of healthy and unhealthy food products. In this study, we focused on the unhealthy snack products. For each food item, participants could indicate their intake on an 8-point scale ranging from “0 days a week” (0) to “7 days a week” (7). To inform participants about which products to consider for each item, text and pictures of the products were provided. The scores for soft drinks were used to obtain the measure for SSBs. Scores for chocolate, cake, and candy bars were summed to measure sweet snack intake. Scores for warm, fried snacks were used to measure savory snack intake. The same procedure was followed by van den Broek et al. [75]. The items measuring sweet and savory snack intake were selected from a validated Dutch FFQ measuring fat intake [77]. This FFQ has shown the expected associations with demographic variables in a previous adolescent population [78]. In line with previous studies [75,78,79], all items assessing the intake of sweet snacks were included. However, as a decade has passed, insights on the beneficial effects of certain foods have changed and we therefore made some modifications to the items used to assess savory snacks. Given that the Dutch Nutrition Centre now states that (low-fat) cheese and (unsalted) nuts are part of a healthy diet, we decided to disregard previously included items on “nuts and peanuts” and on “potato chips, pieces of cheese and sausage” for inclusion in our unhealthy, savory snacks measure [77]. Adolescents were asked to indicate how many days per week (0–7) they consumed this particular product in four different contexts: (1) taken or received from home, to eat or to drink at home or to take away; (2) bought at school, such as from the canteen or the vending machine; (3) bought somewhere else, such as in the supermarket, snack bar, or sports club; (4) received somewhere else, such as at their neighbors’, grandparents’, or friends’ place. In the current study, we focused on food intake taken or received from home, eaten or drunk at home, or taken away from home to eat or drink somewhere else. We aimed to examine associations in this context because it is in this context that FPP have the most influence on adolescent food intake.

### 3.4. Anthropometrics

Adolescents’ height and weight were measured by trained research assistants according to protocol [80]. Body Mass Index (BMI) was calculated as weight in kilograms divided by height in meters squared. We calculated individual age and gender-specific BMI standard deviation scores (z-scores) using a Dutch representative sample of 0–21-year olds as reference [81,82]. Parents self-reported height and weight data was used to calculate parental BMI.

### 3.5. Covariates

As a higher BMI and snacking seems to be more frequent in lower educated youth and in youth with lower educated parents [78,83,84,85], we controlled for parents’ and adolescents’ educational level in our analyses. Additionally, we controlled for other covariates showing a potential association with adolescent snacking and zBMI [62,65,67,86,87,88,89]. As such, the following covariates were added: parents’ and adolescents’ educational level, adolescents’ gender, parental BMI, and adolescent stress.

Parents’ educational level was coded as 1 = primary/high school, 2 = secondary vocational education, 3 = higher professional education/university, following the guidelines of Statistics Netherlands (CBS) [90]. Adolescents’ educational level was coded as 1 = pre-vocational education (‘VMBO’), 2 = higher general secondary education (‘HAVO’), and 3 = pre-university education (‘VWO’). Adolescents’ gender was coded as males = 1 and females = 2. Adolescent stress was measured by asking adolescents to indicate how often they experienced stress at home and at school in the past year on a 4-point Likert scale (rarely, never, most, or all of the time). We used the mean of these two items by summing them and dividing this by two.

### 3.6. Statistical Analyses

Statistical analyses were performed using the PASW 20.0 software package. Descriptive statistics were used (mean, standard deviations, and percentages) to explore population characteristics and to describe the study sample. First, cross-sectional associations between parental stress, FPPs, the snack variables, and the covariates were examined by calculating Pearson’s correlation coefficients. Second, we performed moderated mediation analyses using model 8 of the Hayes PROCESS Macro (V 3.5) [91] in SPSS to explore the mediating role of FPPs in the association between parental general stress and adolescents’ snacking behavior and zBMI, and to investigate whether the associations in the path model vary according to the parent’s gender and weight status. Multiple mediation analyses with parallel mediators using 5000 bootstrap samples were performed. We performed the analyses with parental general stress at baseline as an independent variable and adolescent snacking (SSB intake, candy intake, and snack intake) at follow-up (1 or 1.5 years later) as dependent variables. We entered the 5 FPPs, also measured at follow-up, (autonomy support, coercive control, modeling, healthy structure, snack structure) as mediators in the model. We ran the analyses in an unadjusted model, a model adjusting for baseline snacking behavior, and one adjusted for covariates. We performed an equivalent set of analyses with adolescent zBMI as dependent variable, adjusting for baseline zBMI.

## 4. Results

### 4.1. Cross-Sectional Associations and Descriptives

The total sample consisted of 400 adolescent–parent dyads. Descriptive statistics were used (mean, standard deviations, and percentages) to describe the study sample (see Table 1). There were no significant differences in mean stress levels between fathers and mothers at baseline. Additionally, no significant differences in mean FPPs or mean BMI were found between fathers and mothers.

Pearson’s correlation coefficients between parental stress, FPPs, snack intake, and covariates are presented in Table 2. Parental general stress at baseline was significantly positively correlated with adolescent snacking at follow-up (r = 0.13), and negatively correlated with FPPs autonomy support at follow-up (r = −0.16). No significant correlations were found between parental general stress at baseline and adolescents’ zBMI at follow-up. Of the FPPs, autonomy support was negatively correlated with all snacking variables: SSB intake (r = −0.12), sweet snack intake (r = −0.20), and savory snack intake (r = −0.20) and healthy structure and snack structure were negatively correlated with savory snack intake (r = −0.11) and SSB intake (r = −0.14), respectively. No significant correlations were found between FPPs and adolescents’ zBMI at follow-up. Of the covariates, educational level of the adolescent (r = −0.13) and educational level of the parent (r = −0.13) was negatively correlated with parental general stress at baseline. Additionally, educational level of the adolescent and educational level of the parent was negatively correlated with SSB intake (parent: r = −0.15, adolescent: r = −0.13) and savory snack intake (parent: r = −0.13, adolescent: r = −0.15), but not with sweet snack intake.

### 4.2. Moderating Role of Parental Gender and Parental BMI

Neither parental gender nor parental BMI were found to moderate the link between parental general stress at baseline and FPPs. In addition, the (three-way) interaction terms between parental gender, adolescent zBMI, and the parental stress–food parenting link were also not found to be significant. We therefore performed mediation analysis on the total sample.

### 4.3. Mediating Role of Food Parenting Practices

Of the five FPPs, only autonomy support mediated the association between parental general stress and adolescent savory snack intake and sweet snack intake, though the effects are small (Table 3). Significant indirect effects were observed with autonomy support and the association between general stress and adolescent savory snack intake (b = 0.01, *p* < 0.05, 95%CI = (0.0000, 0.0142)) and between general stress and adolescent sweet snack intake (b = 0.01, *p* < 0.05, 95% CI = (0.0006, 0.0233)), after correction for covariates. The indirect effect represents the portion of the relationship between parental general stress at baseline and adolescent snacking at follow-up that is mediated by autonomy support at follow-up. This effect was also significant in a reduced model without covariates. As can be seen in Table 4, no significant indirect effects were found for any of the FPPs as a mediator in the link between parental general stress at baseline and adolescent zBMI at follow-up, though coercive control was borderline significant as a mediator of this link (b = 0.003, *p* = 0.05, 95% CI = (−0.0010, 0.0089)) in the unadjusted analyses.

## 5. Discussion

Parents who experience high levels of stress may impact child and adolescent snacking behavior and weight development by using less healthy and/or unhealthier FPPs. The present study fills an important gap in the literature by examining whether the prospective link between parental stress and adolescents’ food intake and weight outcome is mediated by FPPs. Studies examining FPPs in adolescents are surprisingly rare anyhow, whereas parents may still importantly determine adolescents’ intake, and adolescence is a vulnerable time period for the development of snacking behaviors and subsequent overweightness. We found that autonomy support mediated the association between parental general stress at baseline and adolescent savory snack and sweet snack intake at follow-up one year to one and a half years later. This effect was not found for SSB intake or the association between parental general stress and adolescent zBMI. We found no differences in fathers or mothers in the link between stress and FPPs. Additionally, parental BMI also failed to moderate this link.

We hypothesized that parental stress would be associated with less beneficial FPPs that, in turn, would be associated with snacking behaviors and a higher zBMI in adolescents. In line with this hypothesis, we found that parents with higher general stress levels showed lower autonomy support, which preceded the development of greater savory snack and sweet snack intake one year to one and a half years later in adolescents. We did not see the associations between general stress at baseline and zBMI at follow-up. Notably, we previously found that maternal general stress at T1 preceded the development of adolescent’s zBMI 6 months later (at T2) [52]. However, this effect was measured over only 6 months of time when adolescents were still in the preadolescent phase (10–12 years). In the current study, we used a follow-up measurement one full year to one and a half years later on. At that follow-up measurement, most adolescents had shifted from the preadolescent to the mid-adolescent stage (14–16 years), with zBMI at follow-up being sensitive enough to examine individual differences over time and the role of parental stress herein (i.e., the zBMI difference score between baseline and follow-up showed a small average increase in zBMI, and a relatively larger standard deviation implying a fair amount of variation in adolescent BMI growth rates). That parental stress no longer preceded zBMI at the longer-term follow-up in the current study might be explained by the fact that other predictors, including pubertal stage, became more important. Thus, though speculative, it may be that pubertal changes during this transition from preadolescent to mid-adolescent stage overruled the effects of most other predictors, explaining why parental stress did not affect their children’s zBMI over a longer period of time.

Notably, of all the FPPs, only autonomy support mediated the link between parental stress and adolescent snacking. We expected that structured FPPs would also mediate the association between parental stress but this was not the case. It is possible that structured FPPs may play a more important role in younger children when rules, setting limits, and a healthy home environment can still shape certain habits. Given adolescents’ emerging desire to become autonomous individuals, parental autonomy support may be especially important for encouraging autonomous food choices during this developmental period, as adolescents increasingly spend less time with their parents and more time with their peers. It is known that parenting that adjusts to the adolescent’s need for increased autonomy can foster independence [53,54], and may encourage adolescents’ learning and development of healthful eating [55]. Though speculating, for stressed parents it may be particularly complicated to guide their adolescent children into becoming autonomous individuals because this a relatively novel parenting practice for them. Up to that time, parents have had ample experience with structured parenting practices and modeling, but less with autonomy supportive practices [92,93]. This might explain why, in particular, autonomy support mediated the link between parental stress and outcome.

Although autonomy support was found to be an important mediator of the parental general stress–adolescent snack intake link, some caution is needed when interpreting the generalizability of the results. Given that our sample consisted of a high percentage of highly educated respondents as well as a high proportion of respondents of Dutch origin, this could possibly have influenced the generalizability of the results. For instance, there is likely to be greater nutrition knowledge in higher educated groups and more time and money available for healthy food parenting [94,95]. Groups that are more ethnically diverse, and in which more lower educated parents and adolescents are represented, could yield different outcomes, specifically as stress and unhealthy behavior are more prevalent in lower SES groups [96]. Additionally, it is known that different forms of general parenting (more authoritarian and more indulgent) are used in lower SES groups [97,98] than in higher SES groups. Though speculating, it is possible that other FPPs rather than autonomy support could mediate the association between parental general stress and snack intake in lower SES groups. For instance, availability has been identified as an important environmental influence among low-income parent/child dyads regarding fruit, vegetable, and sugar-sweetened beverage consumption [99].

No differences were found between fathers and mothers in FPPs being used in general. Additionally, the link between general stress and FPPs was not moderated by parental gender: mothers and fathers did not respond differently in their food parenting when exposed to stress. Previous research has shown that fathers reported significantly more use of coercive food parenting strategies than mothers [100,101]. These strategies include punishment, pressure, emotional feeding, and using food as a reward. Fathers also reported significantly less involvement in autonomy promotion and structure-based food parenting strategies than mothers [100]. Our study did not replicate these findings. This may be explained by the fact that the fathers who participated in our study might be more invested in general parenting and, specifically, food parenting, than fathers who did not fill in a questionnaire. Furthermore, parental weight status was not found to moderate the association between parental stress and FPPs used. We expected, based on the literature, to find differences in structured FPPs and modeling in parents with higher versus lower BMI. We did not find this, possibly because our sample of parents consisted mainly of relatively healthy weight adults.

The current study had several strengths and limitations. One notable strength of this study is that it is, to the best of our knowledge, the first study that prospectively examined the link between parental stress and adolescents’ dietary intake and weight outcomes as mediated by differential FPPs. However, one obvious limitation is that both food parenting and adolescents’ outcomes were measured at the same time. As such, we do not have any insight into the causal order of effects between food parenting, snacking, and weight outcomes. Food parenting may predict children’s eating behaviors and weight gain over time, but FPPs could equally be a parent’s response to child food intake and weight [102]. A few recent studies have attempted to clarify the direction of the associations and whether FPPs are a predictor or a consequence of children’s eating behaviors but the results are mixed [57,103,104]. Both unidirectional and bidirectional associations were found depending on the food parenting practice, eating behavior, and age of the children. Moreover, the size of the mediation effect was quite small, which can also be seen as a limitation. The effect is robust, though it was found in adjusted and unadjusted analyses in a prospective study on parental stress and food parenting versus food intake and weight outcomes in adolescents. The availability of linked parent–adolescent data, adds to the study’s strength. Furthermore, adolescents reported on their own food intake, which can be seen as a strength because parents do not know of everything an adolescent may snack on. However, this can also be seen as a limitation. Dietary reporting bias is possible in self-reported data. In our study sample of relatively highly educated respondents, underreporting of energy intake was possible due to social desirability bias. Although the schools which participated in our study were representative of the Dutch school population [105,106], not every school had the same number of students and participation rates varied across schools and classes, with the highest participation rates in the schools and classes with the highest education levels. Possibly, this is due to the active informed consent procedure that we used: school samples recruited with active parental consent procedures are known to be less diverse and have fewer high-risk participants [3,4]. In line with this, a limitation of this study is that the sample consisted of a high percentage of highly educated respondents, possibly influencing the generalizability of the results.

## 6. Conclusions

To conclude, our findings suggest that parents who experience more general stress use the FPP autonomy support less often, which in turn results in more snack intake of the adolescent between one and one and a half years later. Our study adds to the increasing body of literature regarding FPPs and adolescents’ dietary behaviors, and thus verifies that parental influence on adolescents’ dietary behaviors does not cease to exist once childhood ends. In ecological momentary assessment (EMA) studies, associations can be found between momentary stress and FPPs; further research may, thus, examine if momentary stress has a comparable effect concerning this specific mediator for adolescents versus other age groups. Interventions to enhance resources for parents to deal with stress might help parents in using more autonomy supportive FPPs. Additionally, future researchers should consider interventions to minimize parental stress to promote healthy parent food-related parenting practices. Lastly, interventions that support and educate parents about autonomy supportive parenting practices in relation to their adolescent’s food choices may be helpful in adolescents’ development of healthy eating behaviors.

## Figures and Tables

**Table 1:** Socio-demographic characteristics of the study sample.

	Total Study Sample (*n* = 400 Parents and Adolescents)
Age of the child (years) at baseline; mean (SD)	12.9 (0.62)
Range	11.3–14.8
Age of the child (years) at follow-up; mean (SD)	14.3 (0.61)
Range	12.8–16.3
Gender (% male)	49.1
Gender parent (% male)	30.8
Age of the mother (years) at baseline; mean (SD)	44.6 (4.2)
Range	29.8–57.3
Age of the father (years) at baseline; mean (SD)	47.8 (4.1)
Range	37.8–61.4
**Educational level mother (%)**	
Primary school/high school	10.9
Secondary vocational education (MBO)	35.6
Higher professional education (HBO)	44
University	9.5
**Educational level father (%)**	
Primary school/high school	8.9
Secondary vocational education (MBO)	27.6
Higher professional education (HBO)	41.5
University	22
**Educational level adolescent (%)**	
Pre-vocational education (VMBO)	38.3
Higher general secondary education (HAVO)	10.1
Pre-university education (VWO)	51.6
Adolescent zBMI at baseline; mean (SD)	0.13 (1.04)
Range	−2.90–2.94
Adolescent zBMI at follow-up; mean (SD)	0.42 (1.05)
Range	−2.71–2.87
General stress mother at baseline mean (SD)	8.0 (2.2)
Range	4–15
General stress father at baseline mean (SD)	8.1 (2.3)
Range	4–15
Maternal BMI; mean (SD)	24.8 (4.5)
Range	17.7–43.7
Paternal BMI; mean (SD)	25.7 (3.7)
Range	19.4–36.9
**FPP scores mother**	
Autonomy Support; mean (SD)	4.5 (0.54)
Range	1–5
Coercive Control; mean (SD)	2.2 (1.01)
Range	1–5
Modeling; mean (SD)	3.4 (1.08)
Range	1–5
Healthy Structure; mean (SD)	4.8 (.45)
Range	1–5
Snack Structure; mean (SD)	3.7 (0.88)
Range	1–5
**FPP scores father**	
Autonomy Support; mean (SD)	4.4 (0.69)
Range	1–5
Coercive Control; mean (SD)	2.0 (1.00)
Range	1–5
Modeling; mean (SD)	3.4 (1.20)
Range	1–5
Healthy Structure; mean (SD)	4.7 (0.52)
Range	1–5
Snack Structure; mean (SD)	3.7 (0.94)
Range	1–5

**Table 2:** Correlational associations between adolescent snack intake at follow-up, parental factors at baseline and follow-up, and parental FPPs at follow-up.

	1.	2.	3.	4.	5.	6.	7.	8.	9.	10.	11.	12.	13.	14.	15.	16.	17.
**Adolescent Snack Intake**																	
**1. SSB intake**	1																
**2. Sweet snack intake**	0.30 **	1															
**3. Savory snack intake**	0.29 **	0.38 **	1														
**Parental factors**																	
**4. General Stress baseline**	0.03	0.07	0.13 *	1													
**5. General Stress follow** **-** **up**	0.05	0.08	0.07	0.43 **	1												
**6. Gender parent**	0.13 *	−0.01	0.06	−0.03	0.09	1											
**7. BMI parent**	0.02	−0.10	0.03	0.08	−0.10	−0.10	1										
**FPPs**																	
**8. Autonomy Support**	−0.12 *	−0.20 **	−0.20 **	−0.16 **	−0.11 *	0.08	−0.16 **	1									
**9. Coercive Control**	−0.01	0.07	−0.02	0.08	0.12 *	0.08	0.06	0.02	1								
**10. Modeling**	−0.04	−0.09	0.04	−0.07	−0.08	0.03	−0.12 *	0.25 **	0.15 **	1							
**11. Healthy Structure**	−0.06	0.02	−0.11 *	−0.03	−0.16 **	0.07	−0.23 **	0.33 **	−0.04	0.06	1						
**12. Snack Structure**	−0.14 *	−0.07	−0.06	0.02	0.04	0.03	−0.02	0.27 **	0.01	0.26 **	0.18 **	1					
**Covariates**																	
**13. zBMI adolescent**	−0.08	−0.17 **	−0.06	−0.06	−0.03	0.02	0.23 **	0.06	0.03	−0.00	−0.08	−0.02	1				
**14. Gender adolescent**	−0.20 **	−0.08	−0.13 *	0.02	0.02	−0.05	0.04	−0.03	−0.03	−0.03	−0.04	−0.04	0.12 *	1			
**15. Adolescent stress**	−0.01	0.08	−0.07	0.06	0.12 *	−0.09	−0.11 *	0.08	0.05	−0.02	0.06	−0.06	0.10	0.11 *	1		
**16. Educational level adolescent**	−0.13 *	−0.09	−0.15 **	−0.13 *	−0.10 *	−0.05	−0.06	0.19 **	0.08	0.07	0.20 **	−0.02	−0.09	0.05	0.06	1	
**17. Educational level parent**	−0.15 **	−0.01	−0.13 *	−0.13 **	−0.14 **	−0.10 *	−0.21 **	0.17 **	0.04	0.05	0.22 **	0.06	−0.12 *	0.04	0.13 *	0.36 **	1
**Mean (SD)**	2.91 (2.30)	1.81 (1.30)	1.13 (0.88)	8.0 (2.25)	7.45 (2.25)	30.8% male	25.11 (4.30)	4.51 (.60)	2.16 (1.01)	3.41 (1.11)	4.77 (0.48)	3.73 (.90)	0.42 (1.05)	49.1% male	2.12 (0.70)	51.6% higher 10.1% mid 38.3% lower	56.5% higher 43.5% lower
**Range**	0–7	0–7	0–7	4–15	4–16	−	17.72–43.75	1–5	1–5	1–5	1–5	1–5	−2.71– 2.87	−	1–4	−	−
***n***	376	376	376	400	400	400	397	393	393	393	393	393	366	399	376	376	398

** significant at the 0.01 level (2-tailed). * significant at the 0.05 level (2-tailed). Adolescents’ stress, educational level, gender, zBMI. Correlations for men and women were not significantly different, we therefore present correlations in the total sample.

**Table 3:** Mediation analysis of the link between parental general stress at baseline (X) and adolescent snacking at follow-up (Y) by Food Parenting Practices at follow-up (M).

X	M	Y	A Path (X–M)				B Path (M–Y)				C’ Path (Direct Effect X–Y)				Bootstrap Results for Indirect Effect			
**Adjusted for baseline snack, sweet and SSB consumption**			B	SE	t	*p*	B	SE	t	*p*	B	SE	t	*p*	B	SE	LL95CI	UL95CI
**General stress**	Autonomy Support	Savory snack Intake	−0.04 **	0.01	−3.14	0.002	−0.18 **	0.08	−2.31	0.002	0.04 *	0.02	2.32	0.02	0.01	0.01	0.0090	0.0184
**General Stress**	Coercive Control	Savory snack Intake	0.04	0.02	1.67	0.10	−0.03	0.04	−0.87	0.39	0.04 *	0.02	2.32	0.02	−0.001	0.002	−0.0065	0.0020
**General Stress**	Modeling	Savory snack Intake	−0.04	0.03	−1.47	0.14	0.08 *	0.04	1.99	0.04	0.04 *	0.02	2.32	0.02	−0.003	0.003	−0.0087	0.0011
**General Stress**	Healthy Structure	Savory snack Intake	−0.01	0.01	−0.99	0.32	−0.04	0.09	−0.43	0.66	0.04 *	0.02	2.32	0.02	0.001	0.001	−0.0020	0.0037
**General Stress**	Snack Structure	Savory snack Intake	0.002	0.02	0.09	0.93	−0.01	0.05	−0.16	0.87	0.04 *	0.02	2.32	0.02	−0.000	0.001	−0.0022	0.0020
**General stress**	Autonomy Support	Sweet snack Intake	−0.04 **	0.01	−3.10	0.002	−0.34 **	0.11	−3.08	0.002	0.01	0.03	0.52	0.60	0.01	0.01	0.0032	0.0301
**General Stress**	Coercive Control	Sweet snack Intake	0.04	0.02	1.60	0.11	0.05	0.06	0.76	0.45	0.01	0.03	0.52	0.60	0.002	0.003	−0.0035	0.0090
**General Stress**	Modeling	Sweet snack Intake	−0.04	0.03	−1.45	0.15	−0.06	0.06	−1.04	0.30	0.01	0.03	0.52	0.60	0.002	0.003	−0.0021	0.0088
**General Stress**	Healthy Structure	Sweet snack Intake	−0.01	0.01	−1.03	0.31	0.14	0.13	1.07	0.29	0.01	0.03	0.52	0.60	−0.004	0.003	−0.0078	0.0030
**General Stress**	Snack Structure	Sweet snack Intake	0.003	0.02	0.16	0.87	−0.04	0.07	−0.54	0.59	0.01	0.03	0.52	0.60	−0.0001	0.002	−0.0033	0.0038
**General stress**	Autonomy Support	SSB intake	−0.04 **	0.01	−3.22	0.001	−0.23	0.21	−1.11	0.27	0.03	0.05	0.68	0.49	0.01	0.01	−0.0076	0.0290
**General Stress**	Coercive Control	SSB intake	0.04	0.02	1.70	0.09	−0.11	0.11	−0.96	0.34	0.03	0.05	0.68	0.49	−0.004	0.01	−0.0190	0.0058
**General Stress**	Modeling	SSB intake	−0.04	0.03	−1.51	0.13	0.06	0.11	0.59	0.56	0.03	0.05	0.68	0.49	−0.002	0.01	−0.0141	0.0071
**General Stress**	Healthy Structure	SSB intake	−0.01	0.01	−1.03	0.30	−0.02	0.24	−0.06	0.95	0.03	0.05	0.68	0.49	0.0002	0.004	−0.0074	0.0079
**General Stress**	Snack Structure	SSB intake	0.0003	0.02	0.01	0.99	−0.16	0.13	−1.19	0.23	0.03	0.05	0.68	0.49	0.000	0.004	−0.0090	0.0094
**X**	**M**	**Y**	**A Path (X** **–** **M)**				**B Path (M** **–** **Y)**				**C’ Path (Direct Effect** **X–Y)**				**Bootstrap Results for** **Indirect Effect**			
**Adjusted for baseline snack, sweet and SSB consumption, and covariates ^a^**			B	SE	t	*p*	B	SE	t	*p*	B	SE	t	*p*	B	SE	LL95CI	UL95CI
**General stress**	Autonomy Support	Savory snack Intake	−0.04 *	0.01	−2.64	0.01	−0.16 *	0.08	−2.00	0.04	0.04 *	0.02	2.01	0.04	0.0054	0.0037	0.0000	0.0142
**General Stress**	Coercive Control	Savory snack Intake	0.05	0.02	1.91	0.06	−0.03	0.04	−0.77	0.44	0.04 *	0.02	2.01	0.04	−0.0013	0.0023	−0.0070	0.0025
**General Stress**	Modeling	Savory snack Intake	−0.03	0.03	−1.17	0.24	0.08	0.04	1.99	0.05	0.04 *	0.02	2.01	0.04	−0.0022	0.0025	−0.0081	0.0018
**General Stress**	Healthy Structure	Savory snack Intake	−0.01	0.01	−0.59	0.56	−0.05	0.09	−0.54	0.59	0.04 *	0.02	2.01	0.04	0.0002	0.0014	−0.0021	0.0038
**General Stress**	Snack Structure	Savory snack Intake	−0.004	0.02	−0.22	0.83	−0.02	0.05	−0.45	0.65	0.04 *	0.02	2.01	0.04	0.0000	0.0012	−0.0025	0.0028
**General stress**	Autonomy Support	Sweet snack Intake	−0.03 *	0.01	−2.46	0.01	−0.31 *	0.11	−2.75	0.01	0.002	0.03	0.09	0.93	0.0099	0.0059	0.0006	0.0233
**General Stress**	Coercive Control	Sweet snack Intake	0.04	0.02	1.82	0.07	0.08	0.06	1.29	0.20	0.002	0.03	0.09	0.93	0.0036	0.0037	−0.0021	0.0124
**General Stress**	Modeling	Sweet snack Intake	−0.03	0.03	−1.13	0.26	−0.04	0.06	−0.74	0.46	0.002	0.03	0.09	0.93	0.0013	0.0024	−0.0025	0.0073
**General Stress**	Healthy Structure	Sweet snack Intake	−0.01	0.01	−0.62	0.54	0.10	0.14	0.72	0.47	0.002	0.03	0.09	0.93	−0.0002	0.0018	−0.0036	0.0040
**General Stress**	Snack Structure	Sweet snack Intake	−0.001	0.02	−0.06	0.95	0.04	0.07	0.59	0.56	0.002	0.03	0.09	0.93	0.0001	0.0017	−0.0035	0.0038
**General stress**	Autonomy Support	SSB intake	−0.04 *	0.01	−2.59	0.01	−0.15	0.21	−0.72	0.47	0.01	0.05	0.15	0.88	0.0049	0.0068	−0.0097	0.0181
**General Stress**	Coercive Control	SSB intake	0.05	0.02	1.94	0.05	−0.03	0.11	−0.29	0.77	0.01	0.05	0.15	0.88	−0.0014	0.0061	−0.0154	0.0101
**General Stress**	Modeling	SSB intake	−0.03	0.03	−1.19	0.24	0.05	0.11	0.47	0.64	0.01	0.05	0.15	0.88	−0.0015	0.0042	−0.0118	0.0056
**General Stress**	Healthy Structure	SSB intake	−0.01	0.01	−0.57	0.57	−0.003	0.25	−0.01	0.99	0.01	0.05	0.15	0.88	0.0000	0.0028	−0.0068	0.0056
**General Stress**	Snack Structure	SSB intake	−0.01	0.02	−0.21	0.83	−0.18	0.13	−1.37	0.17	0.01	0.05	0.15	0.88	0.0002	0.0049	−0.0097	0.0112

** significant at the 0.01 level (2−tailed). * significant at the 0.05 level (2−tailed). ^a^ Covariates: parents’ educational level, parental BMI; Adolescents’ stress, educational level, gender, zBMI.

**Table 4:** Mediation analysis of the link between parental general stress at baseline (X) and adolescent z BMI at follow-up (Y) by Food Parenting Practices at follow-up (M).

X	M	Y	A Path (X–M)				B Path (M–Y)				C’ Path (Direct Effect X–Y)				Bootstrap Results for Indirect Effect			
**Adjusted for baseline zBMI**			B	SE	t	*p*	B	SE	t	*p*	B	SE	t	*p*	B	SE	LL95CI	UL95CI
**General stress**	Autonomy Support	zBMI	−0.05 *	0.01	−3.10	0.002	0.07	0.06	1.14	0.25	−0.02	0.01	−1.40	0.16	−0.0032	0.0023	−0.0082	0.0011
**General Stress**	Coercive Control	zBMI	0.05	0.03	1.94	0.05	0.06	0.03	2.01	0.05	−0.02	0.01	−1.40	0.16	0.0031	0.0025	−0.0010	0.0089
**General Stress**	Modeling	zBMI	−0.05 *	0.03	−2.03	0.04	−0.003	0.03	−0.08	0.93	−0.02	0.01	−1.40	0.16	0.0001	0.0019	−0.0038	0.0041
**General Stress**	Healthy Structure	zBMI	−0.02	0.01	−1.36	0.17	−0.07	0.07	−0.98	0.33	−0.02	0.01	−1.40	0.16	0.0011	0.0016	−0.0018	0.0048
**General Stress**	Snack Structure	zBMI	−0.002	0.02	−0.09	0.93	−0.01	0.04	−0.13	0.90	−0.02	0.01	−1.40	0.16	0.0000	0.0008	−0.0015	0.0018
**Adjusted for baseline zBMI and covariates ^a^**																		
**General stress**	Autonomy Support	zBMI	−0.04 *	0.01	−2.56	0.01	0.05	0.06	0.88	0.38	−0.02	0.02	−1.09	0.27	−0.0020	0.0020	−0.0064	0.0015
**General Stress**	Coercive Control	zBMI	0.05	0.03	1.94	0.05	0.06	0.03	1.79	0.07	−0.02	0.02	−1.09	0.27	0.0029	0.0024	−0.0010	0.0085
**General Stress**	Modeling	zBMI	−0.04	0.03	−1.59	0.11	−0.01	0.03	−0.41	0.68	−0.02	0.02	−1.09	0.27	0.0006	0.0016	−0.0025	0.0043
**General Stress**	Healthy Structure	zBMI	−0.01	0.01	−0.75	0.45	−0.09	0.07	−1.27	0.20	−0.02	0.02	−1.09	0.27	0.0008	0.0015	−0.0021	0.0042
**General Stress**	Snack Structure	zBMI	−0.001	0.02	−0.02	0.98	0.01	0.04	0.15	0.88	−0.02	0.02	−1.09	0.27	0.0000	0.0008	−0.0017	0.0018

* significant at the 0.05 level (2-tailed). ^a^ Covariates: parents’ educational level, parental BMI; Adolescents’ stress, educational level, gender, zBMI.

## Data Availability

The datasets generated and analyzed during the current study are not publicly available due to agreements we have made concerning the exchange and use of our data, but are available from the corresponding author (M.K.) on reasonable request.
